# Rheumatism as a cause of cardiac hemangioma: a rare case report and review of literature with special focus on etiology

**DOI:** 10.1186/s12872-023-03241-8

**Published:** 2023-04-21

**Authors:** Ting Xie, Matiullah Masroor, Xuan Chen, Fujin Liu, Jie Zhang, Dayan Yang, Cong Liu, Mei Xiang

**Affiliations:** 1 Department of Cardiac Surgery, Hainan General Hospital, Hainan Affiliated Hospital of Hainan Medical University, Haikou, 570311 China; 2 Department of Cardiovascular Surgery, Union Hospital, Tongji Medical College, Huazhong University of Science and Technology, Wuhan, 430022 China; 3 Department of Cardiothoracic and Vascular Surgery, Amiri Medical Complex, Afshar, Kabul Afghanistan; 4 International College of Nursing, Hainan Vocational University of Science and Technology, Haikou, 570311 China; 5 Department of Pathology, Hainan General Hospital, Hainan Affiliated Hospital of Hainan Medical University, Haikou, 570311 China; 6 Department of Ultrasound Medicine, Hainan General Hospital, Hainan Affiliated Hospital of Hainan Medical University, Haikou, 570311 China; 7 Department of Ultrasound Medicine, Union Hospital, Tongji Medical College, Huazhong University of Science and Technology, Wuhan, 430022 China

**Keywords:** Rheumatism, Hemangioma, Cardiac surgery, Benign heart tumors, Rheumatic heart disease

## Abstract

**Background:**

Cardiac hemangioma is a very rare benign tumor of the heart which accounts for 1–2% of all primary cardiac tumors. Multiple cardiac hemangiomas are even rarer with only three cases published in the literature. Pathologically it can be divided into cavernous hemangioma, capillary hemangioma, arteriovenous hemangioma, mixed-type hemangioma, and so on. At present, the etiology of cardiac hemangioma is not completely clear. In this study, we present multiple cardiac hemangiomas located in the right atrium and discuss the new unreported possible cause (rheumatism) of cardiac hemangioma. This is the fourth case of multiple cardiac hemangiomas in the medical literature and the first time to present rheumatism as the cause of cardiac hemangioma.

**Case presentation:**

A 53-year-old man presented to the clinic with intermittent chest tightness and shortness of breath for 2 years. On echocardiography, multiple soft tissue masses in the right atrium were found. The patient had rheumatic heart disease with severe mitral stenosis and moderate tricuspid regurgitation. Two masses with a diameter of about 20 mm and 15 mm were seen in the right atrium. One mass was located on the inferior margin of the fossa ovalis and the other was adjacent to the inferior vena cava. Both masses were successfully removed surgically. The mitral valve replacement and tricuspid valve plasty were performed at the same time. The postoperative histopathology results confirmed the diagnosis of cavernous hemangioma.

**Conclusion:**

The occurrence of multiple hemangiomas in the heart is possible, especially in the presence of rheumatism. Rheumatism is one of the possible etiologies of cardiac hemangioma. Cardiologists and cardiac surgeons should be aware of its occurrence and should consider cardiac hemangioma as a differential diagnosis especially in rheumatic heart disease patients when they present with soft tissue cardiac masses for accurate management.

**Supplementary Information:**

The online version contains supplementary material available at 10.1186/s12872-023-03241-8.

## Background

Cardiac hemangioma is a very rare benign primary cardiac tumor. It makes up about 1–2% of all primary heart tumors [1]. The first case of the cardiac hemangioma was described by Uskoff in 1893 [2]. A cardiac hemangioma can arise from any of the cardiac layers: endocardium, myocardium, or epicardium (including the pericardium), among them the epicardium is the less common site of origin [3]. Anatomically the most common site for cardiac hemangioma is the atrium [4]. Females are affected slightly more commonly than men [1]. In general, hemangioma includes capillary hemangioma, cavernous hemangioma, arteriovenous hemangioma, mixed type hemangioma, and so on. The most common type is cavernous hemangioma followed by capillary hemangioma [1, 5]. At present, the etiology of cardiac hemangioma is not completely clear, however, it is generally believed that hemangioma is related to genetic factors, acquired infection, trauma, immunity, etc [5]. There may be no specific signs and symptoms associated with cardiac hemangioma but most of the cases published in the literature have circulatory and respiratory symptoms and symptoms are mostly attributed to its anatomical size, location, and mobility [6]. Accurate preoperative diagnosis is hard but most cases are diagnosed on postoperative histopathology results [1, 7]. Echocardiography is used as the first-line diagnostic modality for cardiac tumors but computed tomography (CT) and magnetic resonance imaging (MRI) are used for better visualization and three-dimensional evaluation of tumor as well as tumor relationship with the surrounding structures and surgical feasibility [8–10]. Surgical resection is the mainstay of treatment [1, 7] but treatment with radiotherapy and medication such as corticosteroids, beta-blockers, interferon-α, and anticancer drugs is also available [1]. In this article, we report an interesting case of multiple cardiac cavernous hemangiomas located in the right atrium with concomitant rheumatic heart disease (RHD). Only three cases of multiple cardiac hemangiomas have been published previously [11–13]. At the same time, we did a literature review of cardiac hemangioma with a special focus on etiology. This is the first time that rheumatism has been presented as an etiology of cardiac hemangioma.

## Case presentation

**Fig. 1:**
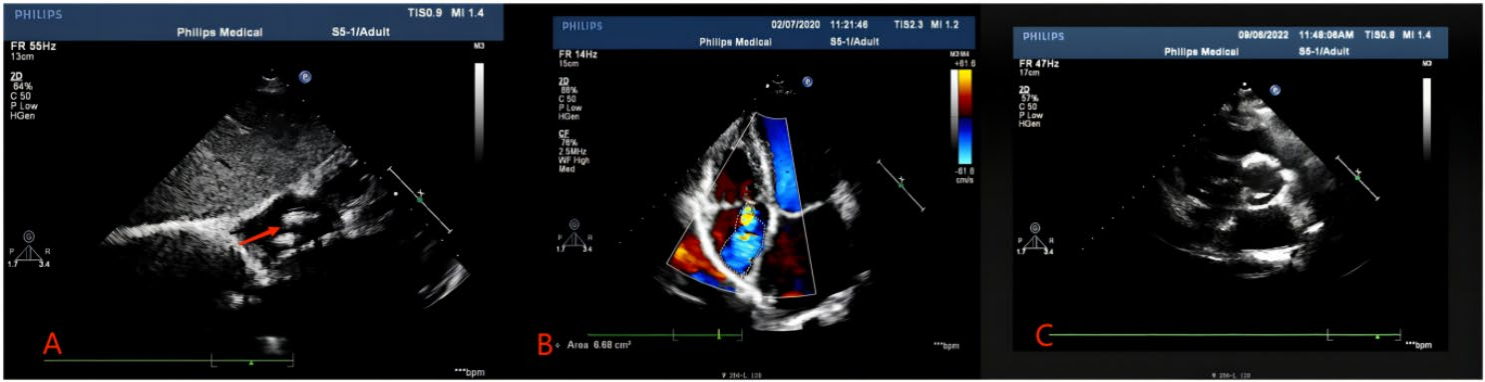
Transthoracic echocardiography images of hemangioma, (**A**) Two hyperechoic masses in the right atrium (red arrow), (**B**) Preoperative echocardiography, (**C**) Postoperative echocardiography

**Fig. 2:**
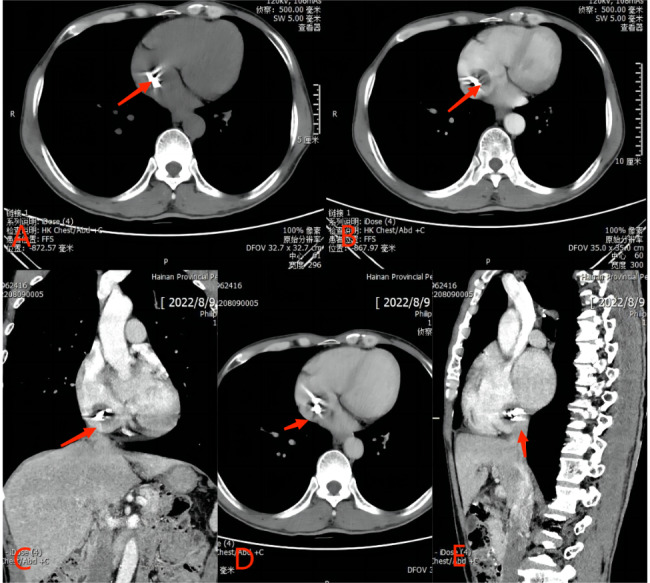
CT examination of hemangioma, (**A**) Abnormal structure in the right atrium by plain CT (red arrow), (**B**) Contrast-enhanced arterial phase, (**C**) Coronal view, (**D**) Contrast-enhanced venous phase, (**E**) Sagittal view

**Fig. 3:**
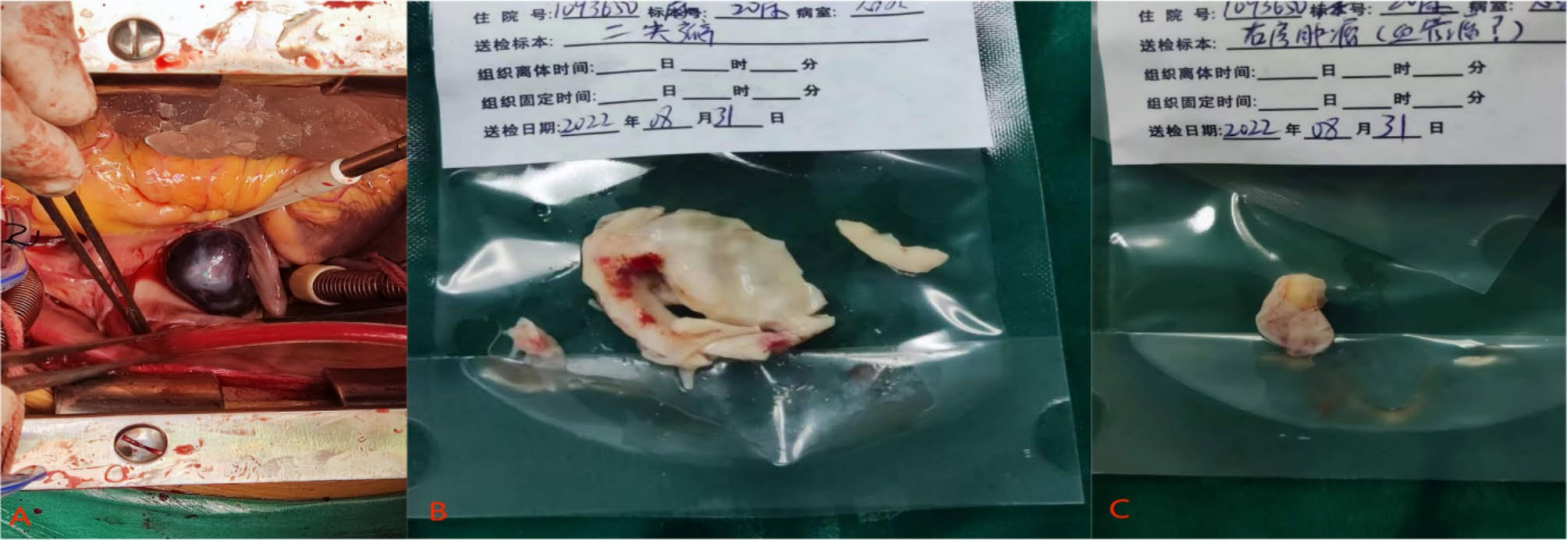
(**A**) Intraoperative view of the hemangioma, (**B**) Specimen of the resected mitral valve leaflets (**C**) Specimen of the hemangioma

**Fig. 4:**
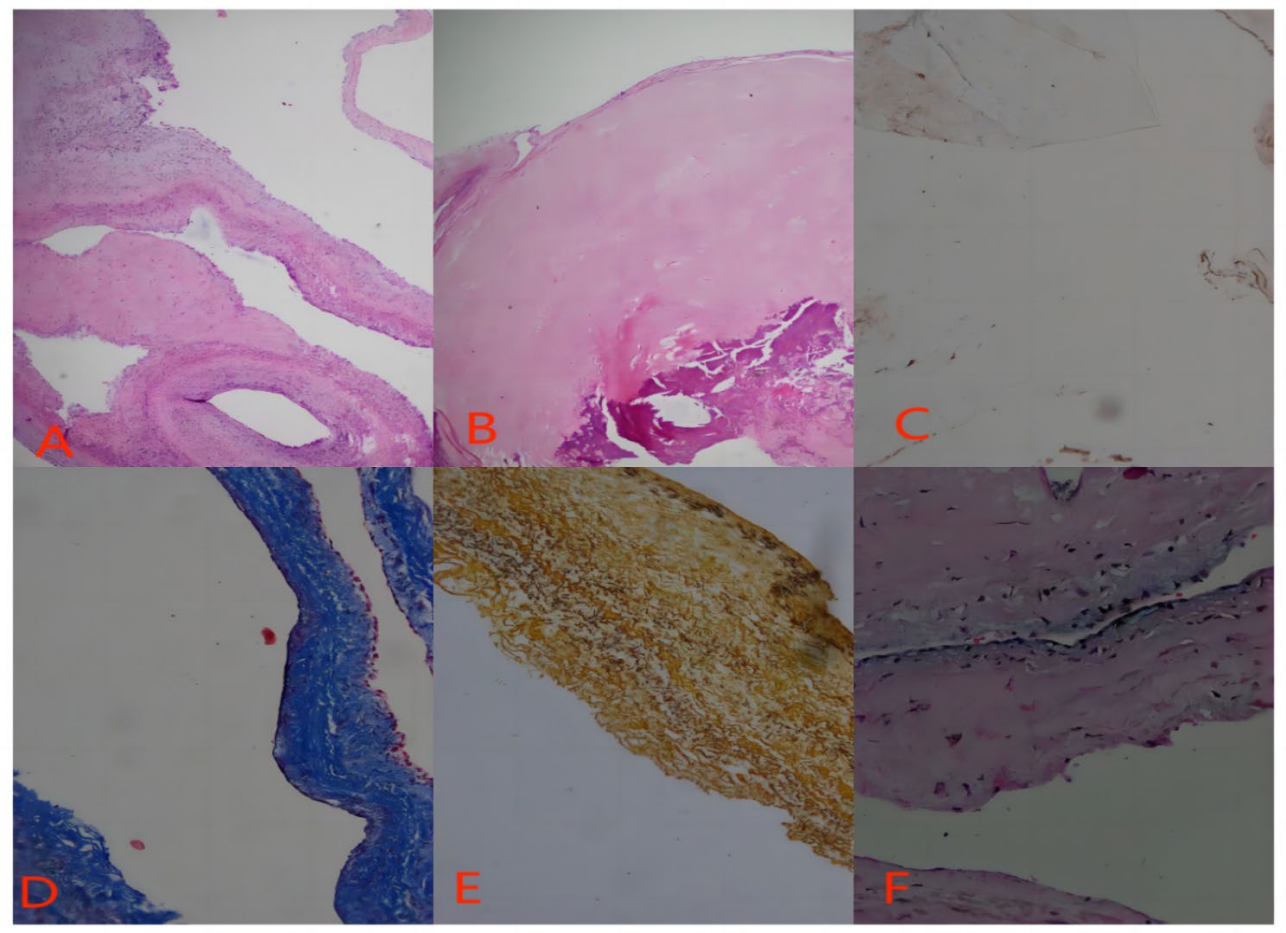
Histopathology of cardiac hemangioma, (**A**) (HE×40): The structure of hemangioma is complete without multinucleated cells, (**B**) Calcification can be seen on HE stain at the base of hemangioma, (**C**) CD34 immunohistochemical staining: CD34+, (**D**) Masson staining: The capsule’s wall structure is complete, (**E**) Reticular fiber staining: The vessel wall structure is complete, (**F**) HE: Degenerative lesions at the base of hemangioma can be seen

This 53-year male presented to our hospital with chief complaints of intermittent chest tightness and shortness of breath for 2 years. He denied any other symptoms. He had no other medical and surgical history except rheumatoid arthritis (RA) for 10 years. He had no smoking or alcoholism history and his family history was unremarkable. On auscultation, diastolic murmur was audible at the 5th intercostal space in the midclavicular line. Transthoracic echocardiography (TTE) showed that the left atrium of the heart was enlarged, and the size of the other heart chambers were approximately normal. The ventricular wall was normal and there was no defect in intra atrial or intraventricular septum. Two hyperechoic masses were seen in the right atrium with the size of about 20 × 15 mm, and 15 × 13 mm respectively, swinging back and forth between the inferior vena cava and the atrial septum with the cardiac cycle. The mitral valve leaflets were thickened and the opening was limited to 10 mm. The valve opening area was 1.3 cm^2^ and a small amount of regurgitation was seen at the mitral valve during the systole. There was moderate tricuspid regurgitation as well. The TTE diagnosis was: (1) The soft tissue masses of the atrial septum in the right atrium (myxomas?); (2) RHD: severe mitral stenosis and mild regurgitation; moderate tricuspid regurgitation (Fig. 1). The plain and contrast-enhanced CT also showed abnormal structures in the right atrium (Fig. 2). After the completion of the preoperative examination, considering the diagnosis of cardiac myxoma, the patients underwent the resection of right atrial masses and mechanical mitral valve replacement with tricuspid valve plasty on cardiopulmonary bypass (Fig. 3). Surgical findings: Tumors were located in the right atrium with a smooth surface and complete capsule. The texture was soft and round with dark red blood in the cavity. One tumor with a diameter of about 20 mm originated from the endocardium in the inferior margin of the fossa ovalis and the base of the tumor was slightly calcified; the other tumor with a diameter of about 15 mm originated from the endocardium adjacent to the inferior vena cava. The mitral valve leaflets were thickened with the fused commissures and narrow valve orifice. The tricuspid valve leaflets were normal. The masses removed during the surgery were sent to pathology and the specimens were stained with HE, masson, CD34 immunohistochemistry, reticular fiber, etc. The pathological results indicate that the tumors were composed of a blood-filled vascular capsule and the structure of the capsule’s wall was complete. No multinucleated cells were found. The capsule’s wall was lined with proliferative vascular endothelial cells. There were few calcifications and degenerative lesions at the base of the tumor (Fig. 4). The histopathology report confirmed the diagnosis of cavernous hemangioma postoperatively. The patient was discharged smoothly one week after the operation. After 6 months of follow-up, the patient was healthy without any symptoms or recurrence of hemangioma.

## Discussion

Similar to other hemangiomas, cardiac hemangioma also arises from the dilatation or abnormal hyperplasia of small capillaries, venules, or arterioles [1, 14]. Benign heart tumors such as myxoma, hemangioma, fibroma, etc. account for almost 75–94% of primary heart tumors. Hemangioma is one of the rarest among these benign tumors which accounts for 1–2% of all primary cardiac tumors [1]. Hochberg and Robinson for the first time in 1950 removed the cardiac hemangioma in history [15]. Miao　et al [1] counted 67 cases of atrial hemangiomas between 1996 and 2018. 62.7% of these cases were in the right atrium and 35.8% of cases were in the left atrium. In one particular case, a hemangioma crossed the left and right atrium (1.5%). A study by Li et al. [16] found that atrial hemangioma accounts for 47% of all cardiac hemangiomas. In our case, two hemangiomas were found in the right atrium. To our knowledge, this is the fourth report that presents multiple hemangiomas in the heart [11–13]. As explained above the most common form of cardiac hemangioma is cavernous hemangioma. The pathology of our sample showed that the hemangioma was blood-filled in a vascular capsule. The histological structure of the capsule wall was normal, without multinucleated cells. The capsule wall was lined with proliferative vascular endothelial cells. There were few calcifications and degenerative lesions at the base of the hemangioma. Therefore, it was considered a cavernous hemangioma.

The etiology of hemangioma is not completely clear [17]. It is generally believed that the following factors may be the cause of hemangioma. (1) Abnormal development of vascular tissue: abnormal development of vascular tissue stimulated during embryonic development; (2) Genetic factors: In recent years,　studies have proved that cavernous hemangioma is an autosomal dominant genetic disease with incomplete dominance; (3) Damage: routine radiotherapy, infection, trauma, surgery, hemorrhage, inflammation, and immune reaction lead to local tissue deformation and necrosis, and the surrounding blood vessels dilate resulting in regional hemangioma formation. Cardiac hemangioma sometimes occurs at the same time with other heart diseases. This patient had cardiac hemangiomas with RHD. Rheumatic disease is an autoimmune and inflammatory disease that can affect every organ including the heart. The cardiac manifestation of this disease is not only because of the specific cardiovascular risk factors in these patients but also related to chronic inflammation and autoimmunity. Any cardiac structure including the endocardium, myocardium, pericardium, heart valves, coronary vessels, and conduction system can be affected by systematic autoimmunity and inflammation [18]. In patients with RHD, inflammation and immune reactions repeatedly occur. We infer that the reason for the formation of cardiac hemangioma is that, after local necrosis of cardiac tissue caused by rheumatism, the blood vessels dilate to form a cavity, and the surrounding blood vessels dilate to form hemangioma. If so, we suspect that hemangioma with RHD not only occurs in the atrium but also may occur in other parts of the heart. Up to now, less than 300 cases of cardiac hemangiomas have been reported. We found that in most cardiac hemangioma published papers, the rheumatism history is not mentioned. Because rheumatism is not the known cause of hemangioma, so even if rheumatism is concomitantly present with cardiac hemangioma, it may be ignored during publication. On the other hand, it is also possible that patients undergo hemangioma resection without proper history and workup for rheumatism and the diagnosis of rheumatism may miss. But it is worth mentioning that there can be other causes of hemangioma in the absence of rheumatism. Fortunately, to support our idea, there are also few reports of hemangioma with RHD in the literature, such as left atrial hemangioma [19], hemangioma of the mitral valve [20, 21], and pericardial hemangioma [3]. It is noteworthy that hemangiomas are occasionally found in the synovial joint of the knee [22, 23]. Like cardiac hemangioma the literature does not mention the history of rheumatism in patients with synovial joint hemangioma which may be the cause of hemangioma. RHD and arthritis are common diseases in developing countries, so we have a reason to believe that rheumatism causes RHD and arthritis, and can also cause hemangioma. We believe rheumatism is one of the unreported etiologies of cardiac hemangioma but need more evidence to support this idea.

Cardiac hemangioma may be asymptomatic and could only be diagnosed on regular health checkups [24]. Symptomatic patients usually have circulatory and respiratory symptoms. It may present with various clinical presentations ranging from simple cough, chest tightness, shortness of breath, palpitation, decrease exercise intolerance, and pleural effusion, to complex presentation such as syncope, angina, conduction abnormalities, arrhythmias, systemic embolic events, systemic congestion, cardiac failure, and even sudden death [1, 8, 16, 25, 26]. The clinical presentation usually depends on the hemangioma’s location, size, growth rate, relationship and infiltration of the adjacent structures, patient’s sex, age, and so on [1, 16].

Precise preoperative diagnosis is very hard to make and most cases are diagnosed on postoperative histopathology. Echocardiography is one of the basic investigations for hemangioma like all other cardiac tumors. It can provide some essential information about the tumor such as tumor size, mobility, and relationship with relevant structures [8] but some advanced investigation tools such as MRI and CT can further clarify the location and three-dimensional view of the tumor which subsequently helps in surgical decision making [9, 10]. Some reports found PET CT a promising diagnostic tool for cardiac hemangioma [27]. Seifert et al. reported a case that showed the signs of malignancy by conventional CT and MRI but FDG PET/CT accurately diagnosed the case as capillary hemangioma [28]. Coronary angiography can help detect the feeding artery and the extent of vascularization of the tumor as well as the evaluation of the native coronary arteries as a reference for the surgery. The tumor blush sign is a common sign found in most cases of atrial hemangioma [1].

There are few treatment options available for cardiac hemangioma but because of the rarity of the condition, there is no consensus on which treatment should be adopted. Based on the available literature which comes from the case reports and case series, the mainstay of treatment is complete or partial surgical resection of the tumor [1, 16]. Because the natural history and hemodynamic consequences of cardiac hemangioma are very unpredictable, therefore surgery is usually recommended for symptomatic patients [29]. However, there are some reports of treating cardiac hemangioma with radiotherapy, and medications like beta blockers (propranolol), corticosteroids, interferon-α, and some anticancer drugs (vincristine, cyclophosphamide, rapamycin, bevacizumab) [30–33]. Wang et al. reported an unresectable case of hemangioma successfully treated with radiotherapy [33]. Even though some nonsurgical treatment options are available, some surgeons still choose to perform surgery for complex hemangiomas. Novitzky et al. published the first case of cardiac autotransplantation for cardiac hemangioma. In their case, it was not possible to resect the hemangioma in situ, so they explanted the heart, resected the hemangioma completely, and reimplanted the heart [25]. We believe cardiac autotransplantation may be an acceptable surgical option when resection in situ is not possible, but this procedure is not necessary in most cases. We also believe cardiac hemangioma caused by rheumatism does not need different treatment, and surgery will remain the standard treatment option. Targeting rheumatism at this stage would not affect the course of hemangioma because pathology has already occurred and the best option would be to completely resect. Though proper management of rheumatism to control recurrent inflammation and autoimmunity at the early stages may prevent future hemangioma formation.

.

## Conclusion

Cardiac hemangioma is an extremely rare disease and is often solitary but multiple cardiac hemangiomas can possibly occur especially in the presence of rheumatism. We believe that rheumatism is one of the etiologies of cardiac hemangioma. Therefore cardiologists and cardiac surgeons should be aware of its occurence and should consider cardiac hemangioma as a differential diagnosis if soft tissue masses are seen in the heart, especially in RHD patients. Surgery is the mainstay of treatment and patients undergoing surgical resection have good outcomes.

## Electronic supplementary material

Below is the link to the electronic supplementary material.

Supplementary Material 1

## Data Availability

All data generated and analyzed during this study are included in this published article.

## Abbreviations

CT: Computed tomography
MRI: Magnetic resonance imaging
RHD: Rheumatic heart disease
RA: Rheumatoid arthritis
TTE: Transthoracic echocardiography
