# Supplementary material for: Rheumatism as a cause of cardiac hemangioma: a rare case report and review of literature with special focus on etiology
Source: BMC Cardiovasc Disord. 2023 Apr 21;23:203. doi: 10.1186/s12872-023-03241-8 (PMC10122334; doi:10.1186/s12872-023-03241-8)
Supplement: Supplementary file 1 — Supplementary Material 1 [file 12872_2023_3241_MOESM1_ESM.pdf]

CARE case report guidelines

CARE Checklist of information to include when writing a case report

(cc) BY-NC-ND

Topic	Item	Checklist item description	Reported on Line
Title	1	The diagnosis or intervention of primary focus followed by the words "case report"	1,2
Key Words	2	2 to 5 key words that identify diagnoses or interventions in this case report, including "case report"	67,68
Abstract (no references)	3a	Introduction: What is unique about this case and what does it add to the scientific literature?	48-51
	3b	Main symptoms and/or important clinical findings	52,58
	3c	The main diagnoses, therapeutic interventions, and outcomes	59-61
	3d	Conclusion—What is the main "take-away" lesson(s) from this case?	62-66
Introduction	4	One or two paragraphs summarizing why this case is unique (**may include references**)	72-92
Patient Information	5a	De-identified patient specific information.	97
	5b	Primary concerns and symptoms of the patient.	98
	5c	Medical, family, and psycho-social history including relevant genetic information	99
	5d	Relevant past interventions with outcomes	
Clinical Findings	6	Describe significant physical examination (PE) and important clinical findings.	99-112
Timeline	7	Historical and current information from this episode of care organized as a timeline	NA
Diagnostic Assessment	8a	Diagnostic testing (such as PE, laboratory testing, imaging, surveys).	100,111
	8b	Diagnostic challenges (such as access to testing, financial, or cultural)	NA
	8c	Diagnosis (including other diagnoses considered)	109-111
	8d	Prognosis (such as staging in oncology) where applicable	NA
Therapeutic Intervention	9a	Types of therapeutic intervention (such as pharmacologic, surgical, preventive, self-care)	113-114
	9b	Administration of therapeutic intervention (such as dosage, strength, duration)	NA
	9c	Changes in therapeutic intervention (with rationale)	NA
Follow-up and Outcomes	10a	Clinician and patient-assessed outcomes (if available)	
	10b	Important follow-up diagnostic and other test results	132,1333
	10c	Intervention adherence and tolerability (How was this assessed?)	132,133
	10d	Adverse and unanticipated events	132,133
Discussion	11a	A scientific discussion of the strengths AND limitations associated with this case report	168-181
	11b	Discussion of the relevant medical literature **with references**.	135-200
	11c	The scientific rationale for any conclusions (including assessment of possible causes)	188-199
	11d	The primary "take-away" lessons of this case report (without references) in a one paragraph conclusion	200-204
Patient Perspective	12	The patient should share their perspective in one to two paragraphs on the treatment(s) they received	Done
Informed Consent	13	Did the patient give informed consent? Please provide if requested	Yes ■ No □
